# The PER (Preoperative Esophagectomy Risk) Score: A Simple Risk Score to Predict Short-Term and Long-Term Outcome in Patients with Surgically Treated Esophageal Cancer

**DOI:** 10.1097/MD.0000000000002724

**Published:** 2016-02-18

**Authors:** Matthias Reeh, Johannes Metze, Faik G. Uzunoglu, Michael Nentwich, Tarik Ghadban, Ullrich Wellner, Maximilian Bockhorn, Stefan Kluge, Jakob R. Izbicki, Yogesh K. Vashist

**Affiliations:** From the Departments of General, Visceral and Thoracic Surgery (MR, JM, FGU, MN, TG, MB, JRI, YKV) and Department of Intensive Care (SK), University Medical Centre Hamburg-Eppendorf, University of Hamburg, Hamburg; and Departments of General, Visceral and Thoracic Surgery (UW), University Hospital Schleswig-Holstein, Campus Lübeck, University of Lübeck, Lübeck, Germany.

## Abstract

Esophageal resection in patients with esophageal cancer (EC) is still associated with high mortality and morbidity rates. We aimed to develop a simple preoperative risk score for the prediction of short-term and long-term outcomes for patients with EC treated by esophageal resection.

In total, 498 patients suffering from esophageal carcinoma, who underwent esophageal resection, were included in this retrospective cohort study. Three preoperative esophagectomy risk (PER) groups were defined based on preoperative functional evaluation of different organ systems by validated tools (revised cardiac risk index, model for end-stage liver disease score, and pulmonary function test). Clinicopathological parameters, morbidity, and mortality as well as disease-free survival (DFS) and overall survival (OS) were correlated to the PER score.

The PER score significantly predicted the short-term outcome of patients with EC who underwent esophageal resection. PER 2 and PER 3 patients had at least double the risk of morbidity and mortality compared to PER 1 patients. Furthermore, a higher PER score was associated with shorter DFS (*P* < 0.001) and OS (*P* < 0.001). The PER score was identified as an independent predictor of tumor recurrence (hazard ratio [HR] 2.1; *P* < 0.001) and OS (HR 2.2; *P* < 0.001).

The PER score allows preoperative objective allocation of patients with EC into different risk categories for morbidity, mortality, and long-term outcomes. Thus, multicenter studies are needed for independent validation of the PER score.

## INTRODUCTION

Cancer of the esophagus is 1 of the 10 most newly diagnosed cancers worldwide. Surgery is still the only curative therapy option for esophageal cancer (EC).^1^ The long-term survival is poor. The mortality and morbidity associated with esophagectomy for EC, even in high-volume centers, remain high.^2^ However, prediction of perioperative outcome is essential not only for eligible patient selection but also for treatment strategy. Predictive factors for perioperative mortality have been reported previously including sex, age, and comorbidities, as well as experience of the operating surgeon, hospital volume, and neoadjuvant therapy.^3,4^ Accurate preoperative risk assessment of patients with EC is the most promising way to reduce mortality and morbidity and provides a tool to compare the quality of care between different institutions. Several authors reported on risk prognostic models and the use of normograms in EC surgery.^5–7^ Several scores were developed based on the models for perioperative mortality, like the Bartels score^5^ and the Physiological and Operative Severity Score for the enumeration of Mortality adjusted for esophagogastric surgery (O-POSSUM).^6^ The reported risk models were restricted to predict mortality or morbidity or required special tests (e.g., the aminopyrine breath test in Bartels score). Other scores failed to predict mortality correctly, like the O-POSSUM.^7^ Indeed, no predictive model has been developed to assess the perioperative risk and long-term outcome for patients undergoing resection for EC. A standardized preoperative risk-assessment tool is still missing. Such a score should be easy to use and based on simple tests that are globally available in daily clinical work.

The aim of this study was to develop a simple risk score based on routine diagnostic work-up that is able to predict the short-term outcome (mortality and morbidity) as well as the long-term survival of patients undergoing esophageal resection for EC.

## METHODS

The study was approved by the Medical Ethical Committee of Hamburg. Between 1994 and October 2007, a total of 540 patients underwent esophageal resection for EC at the Departments of General, Visceral and Thoracic Surgery of the University Medical Center Hamburg-Eppendorf, Germany. Only patients who had a complete resection (R0) and had histologically proven EC were included in the study. Informed consent was obtained from all patients before including them in a prospective database. All patients had a detailed preoperative assessment of their general health condition and organ function evaluation. Routine tumor staging included esophagogastroduodenoscopy, computed tomography, and blood tests. Operating technique was dependent upon tumor location. Until 1999 our primary approach was a transhiatal (TH) resection with a collar anastomosis, whereas from 2000 onward we primarily performed a thoracoabdominal (TA) resection with a high-intrathoracic anastomosis. Perioperative mortality was defined as 30-day posthospital discharge mortality. The perioperative morbidity included any type of medical or surgical complication appearing within the hospital stay or leading to rehospitalization within 30 days after discharge. Clinical follow-up data were obtained by studying the patients’ clinical charts and by contacting them on an out-patient basis or by phone.

### The Preoperative Esophagectomy Risk Score

The main condition for the development of the preoperative esophagectomy risk (PER) score was simplicity. We focused on diagnostic parameters preoperatively available in a routine diagnostic work-up. Furthermore, we evaluated validated diagnostic tools to assess different organ systems. The composition of the PER score is depicted in Table 1. The revised cardiac risk index (RCRI), the model for end-stage liver disease (MELD) score, and the pulmonary function test (PFT) were chosen as the defining parameters of the PER score. For the RCRI, the preoperative medical history of each patient was studied and each variable (high-risk surgery, history of ischemic heart disease, history of congestive heart failure, history of cerebrovascular disease, insulin-dependent diabetes mellitus, and preoperative serum creatinine >2.0 mg/dL) was assigned a point.^8^ Each point was transferred to the PER score. The MELD was developed to predict the mortality of patients awaiting liver transplantation but has also been correlated to outcomes in nonhepatic surgery.^9,10^ The MELD score is based on the following parameters: serum creatinine, bilirubin, and international normalized ratio for prothrombin time (INR) (MELD = 3.78 × ln[serum bilirubin (mg/dL)] + 11.2 × ln[INR] + 9.57 × ln[serum creatinine (mg/dL)] + 6.43).^11^ The MELD score subgrouping was based on the upper-limit values of the particular parameter (creatinine, INR, and bilirubin) predefined at our institution. A MELD score higher than 8 points was assigned 2 points. The vital capacity (VC) and the forced expiratory volume in 1 s (FEV_1_) were used to classify patients in to 3 different pulmonary function groups. The cutoff values to define the 3 different subgroups with good, moderate, or poor pulmonary function were based on data published in the literature. The cutting values are given in Table 1.

**TABLE 1:**
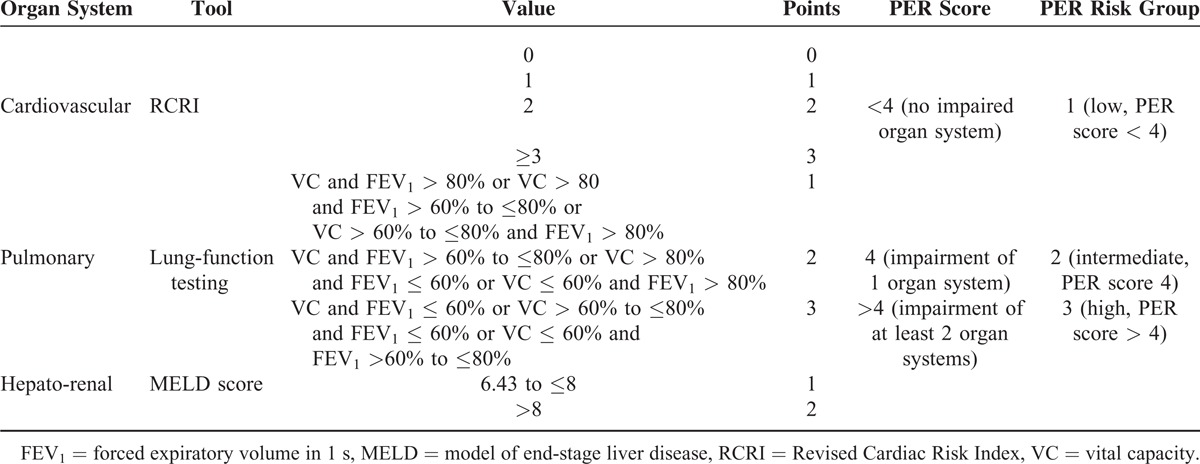
Preoperative Esophagectomy Risk (PER) Score Design and Classification

Assessment of the different organ systems resulted in risk points that were summed to PER score groups. If none of the organ systems was impaired the patient was assigned to the PER group 1. Moderate impairment of 1 organ system resulted in PER group 2 classification and moderate impairment of 2 or more organ systems or severe impairment of 1 organ system directed patients to PER group 3 (see Table 1).

### Characterization of the Study Population

Five hundred forty patients underwent esophageal resection at our institution between 1994 and 2007. Forty-two patients received resection for diseases different from EC, were lost to follow-up, or had incomplete data and were therefore excluded from this study. Complete data and follow-up were available for 498 patients. All 498 patients were surgically treated for EC and had histologically proven EC. None of the patients received neoadjuvant chemotherapy or chemoradiotherapy. The median age of the study population was 63.2 years ranging from 34.5 to 85.2 years. There were 393 (78.9%) males and 105 (21.1%) females. Table 2 depicts the tumor-specific patient characteristics of the entire study population.

**TABLE 2:**
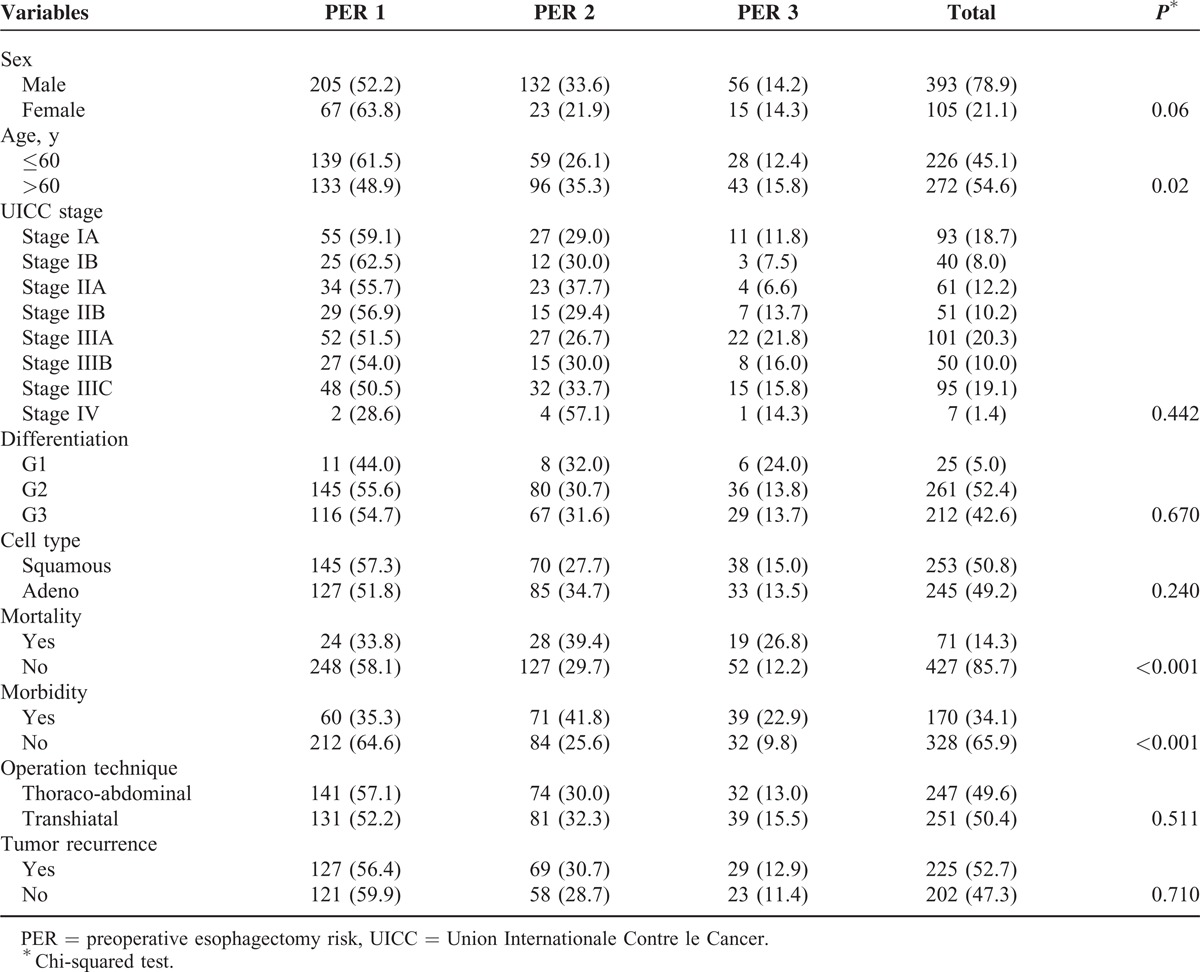
Patient Characteristics and Correlation of the PER Score With Clinicopathological Parameters

### Statistical Analysis

For statistical analysis, SPSS for Windows (version 20.0; IBM Corporation, Armonk, NY) was used. The chi-squared test was used to analyze correlations between clinicopathological parameters and PER score groups. To evaluate the prognostic significance of the PER score for perioperative morbidity and mortality, univariate and multivariate logistic regression analyses were performed, and an odds ratio (OR) with 95% confidence interval (95% CI) was calculated. Survival curves of the patients were plotted using the Kaplan–Meier method and analyzed using the log-rank test. Univariate and multivariate Cox regression analyses were performed to determine the hazard ratio (HR) of different variables for overall survival (OS). Significant statements refer to *P* values of 2-tailed tests that were <0.05.

## RESULTS

The majority of the patients belonged to the low-risk group PER 1 (54.6%), 31.1% were classified in the intermediate-risk group PER 2, and 14.3% were assigned to the high-risk group PER 3. Overall, perioperative mortality accounted for 14.3% and perioperative morbidity for 34.1% of the entire study population.

### Relation of the PER Score With Clinicopathological Parameters and Perioperative Outcome

Table 2 shows the results of the correlation analyses of the PER score groups to clinicopathological parameters. A highly significant correlation was found between the PER score and perioperative outcome in terms of mortality and morbidity. Almost 50% of the patients in the PER 2 and PER 3 groups developed complications in the postoperative course, and almost 18% of the patients in the PER 2 group and 27% in the PER 3 group died perioperatively (*P* < 0.001 each, Table 2).

In the univariate analysis, age and PER score were the only variables reaching statistical significance for perioperative mortality (data not shown). Both parameters were entered into a logistic regression model to evaluate the prognostic significance for perioperative mortality in patients undergoing surgical resection for EC. The PER score was found to be an independent factor for perioperative death with an OR of 2.3 in the PER 2 group and 3.8 in the PER 3 group compared to PER 1 patients (Table 3). For perioperative morbidity, PER score and operating technique were the only significant variables in the univariate analysis. The logistic regression analysis revealed a 3-times higher risk for PER 2 and almost 5-times higher risk for PER 3 of developing a complication in the postoperative course compared to PER 1 patients (Table 3). Patients undergoing TH resection, however, had an almost 50% decrement in the probability of postoperative complications in contrast to patients with TA resection. We found the increase in the PER group from 1 to 3 was paralleled by a progressive increase in the mortality and morbidity rate. The aforementioned correlation between the PER score and the presence of lymph-node metastasis as well as the prognostic significance of the PER score for perioperative mortality and morbidity remained evident also after stratification of the study population to underlying histology (adenocarcinoma [AC] vs squamous cell carcinoma [SCC]) and the type of resection performed (TA vs TH) (data not shown).

**TABLE 3:**
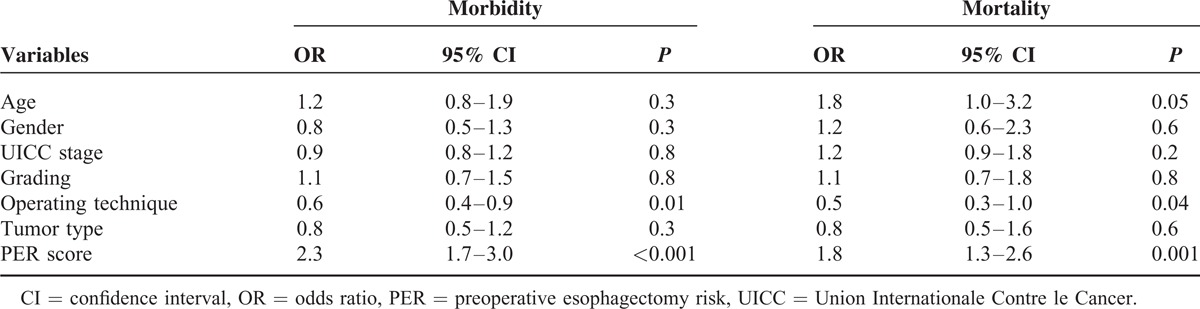
Multivariate Analysis for Perioperative Outcome

### The PER Score and Oncological Outcome

**FIGURE 1:**
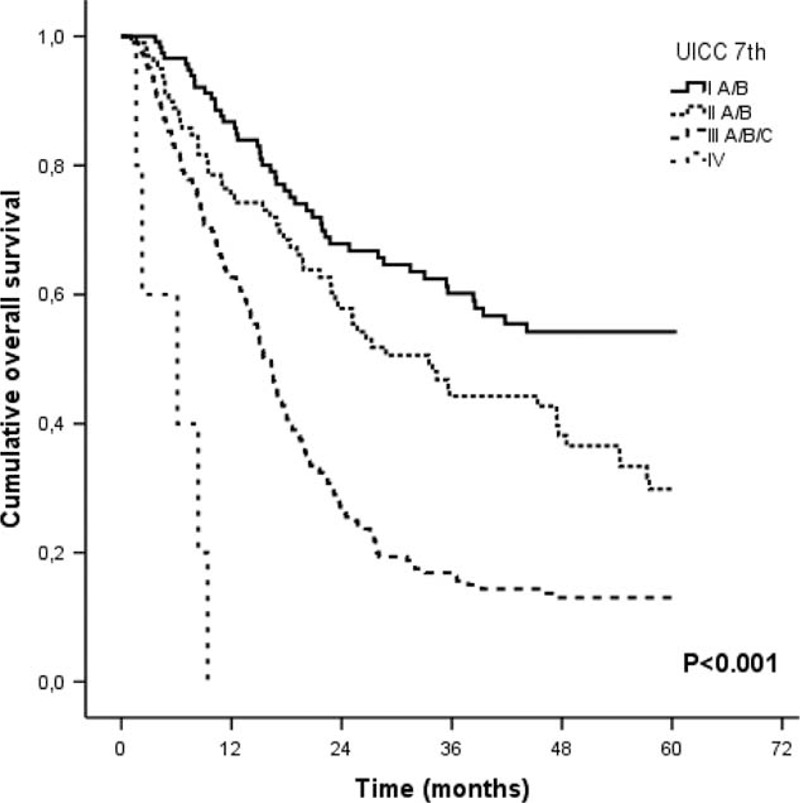
Overall survival according to the 7th edition of UICC classification. UICC = Union Internationale Contre le Cancer.

The median follow-up time was 39.1 months. To verify our study group was representative of patients with EC, we calculated the OS according to the 7th edition of the Union Internationale Contre le Cancer (UICC).^12^ The OS was found to be dependent upon UICC stage and comparable to the data published by other groups (Figure 1).^13,14^

**FIGURE 2:**
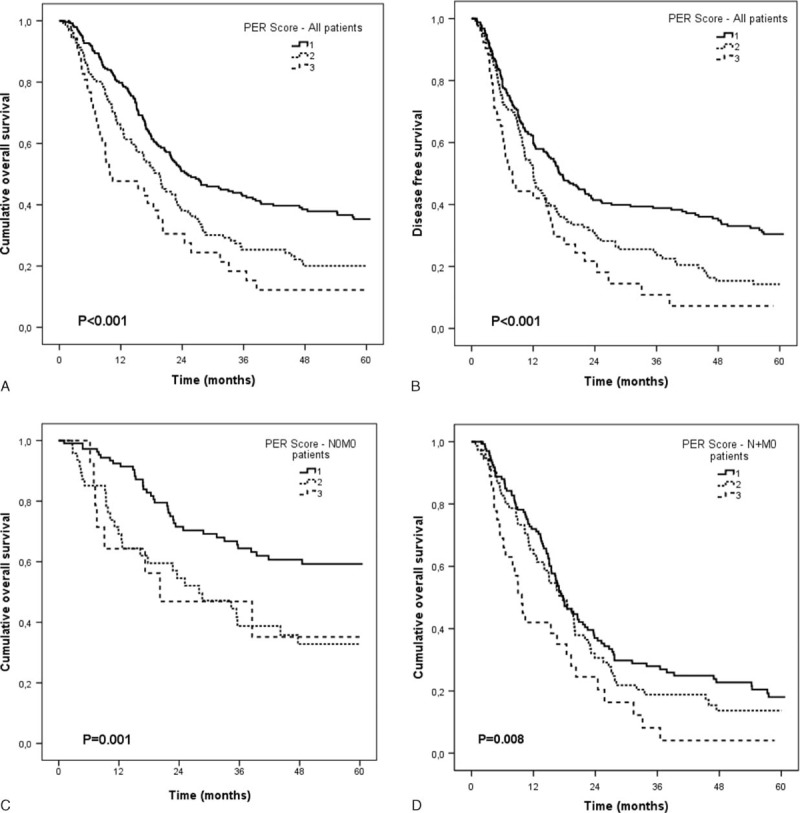
Outcomes of EC patients according to the different preoperative esophagectomy risk (PER) groups. Cumulative overall survival (A) and disease-free survival (B) of all patients according to the PER score. Cumulative overall survival of N0M0 (C) and N + M0 (D) patients according to the PER score. EC = esophageal carcinoma.

Patients who died perioperatively were excluded from the survival analysis. The PER score did not correlate with tumor recurrence (Table 2). However, in the entire study population, a significant association between the PER score and disease-free survival (DFS) was evident (Figure 2B). This association could not be confirmed in subanalyses performed for histology and operating technique (data not shown).

In contrast, a strong correlation was found between the PER score and OS. The median OS of the study population was 21.6 months (CI: 19.1–24.2). The increase in the PER groups was paralleled by a progressive decrease in OS. The median OS in PER group 1 was the longest, with 24.8 months (CI: 18.7–30.9) compared to the shortest survival in PER group 3, with only 9.9 (CI: 1.8–17.9) months. PER 2 patients represented an intermediate group, with a survival of 19.5 months (CI: 15.8–23.2) (Figure 2A, *P* < 0.001).

In our study population, 299 (60%) of the patients were found positive for regional lymph-node metastasis and 92 (18.5%) patients had distant metastases. Patients with positive lymph nodes and distant metastases in EC are known to have extremely poor survival rates, and this may be considered a bias in the correlation between the PER score and the oncological outcome. Hence, we performed survival subanalyses between the PER score and the presence or absence of lymph-node and distant metastases separately. In lymph-node negative patients without distant metastases (N0M0) the mean OS was 43.7 months (CI: 40.3–47.1, median not yet reached). A significant survival difference was evident between the different PER groups (Figure 2C). However, since the presence of lymph-node metastases correlated significantly with the PER score, only 12 patients in the N0M0 cohort belonged to the PER group 3, and the survival analysis was therefore limited to make a concise statement for PER 3 patients in this subgroup (Figure 2C). In lymph-node positive patients without distant metastases (N + M0), a similar pattern of OS to N0M0 patients was evident (Figure 2D). The median OS accounted for 18 months (CI: 15.5–20.5). An increase in PER group was paralleled by a significant stepwise decrease in OS. Patients in PER 3 displayed the poorest OS in concordance with the positive correlation to the presence of lymph-node metastases in this subcohort (Figure 2D). The median OS for PER 1 and PER 2 was 13.4 and 8.6 months, respectively, in contrast to 6.2 months for PER 3 patients (*P* = 0.005).

Since 1 may argue that the oncological outcome may be dependent upon underlying histology or the operating technique, we performed survival-stratified subanalyses in the study population according to the underlying cell type (AC and SCC) and operative procedure (TH and TA resection). In the subanalyses of patients undergoing TA and TH, PER 1 presented the longest (23.8 and 27.2 months, respectively) and PER 3 the poorest (16.6 and 9.9 months, respectively) OS (*P* = 0.002 each). In the subanalyses of patients with SCC and AC, PER 1 presented the longest (22.9 and 27.7 months, respectively) and PER 3 the poorest (9.7 and 10.4 months, respectively) OS (*P* < 0.001 and *P* = 0.008, respectively).

Univariate and multivariate analyses according to the Cox regression hazard model using age, sex, tumor size, presence of lymph-node and distant metastases, tumor differentiation, histology, operative technique, and the PER score were performed. The PER score was found to be an independent prognostic factor of survival of EC, with a HR of 1.4 (95% CI: 1.1–1.9) for PER 2 and 2.3 (95% CI: 1.6–3.4) for PER 3 compared to PER 1 (Table 4).

**TABLE 4:**
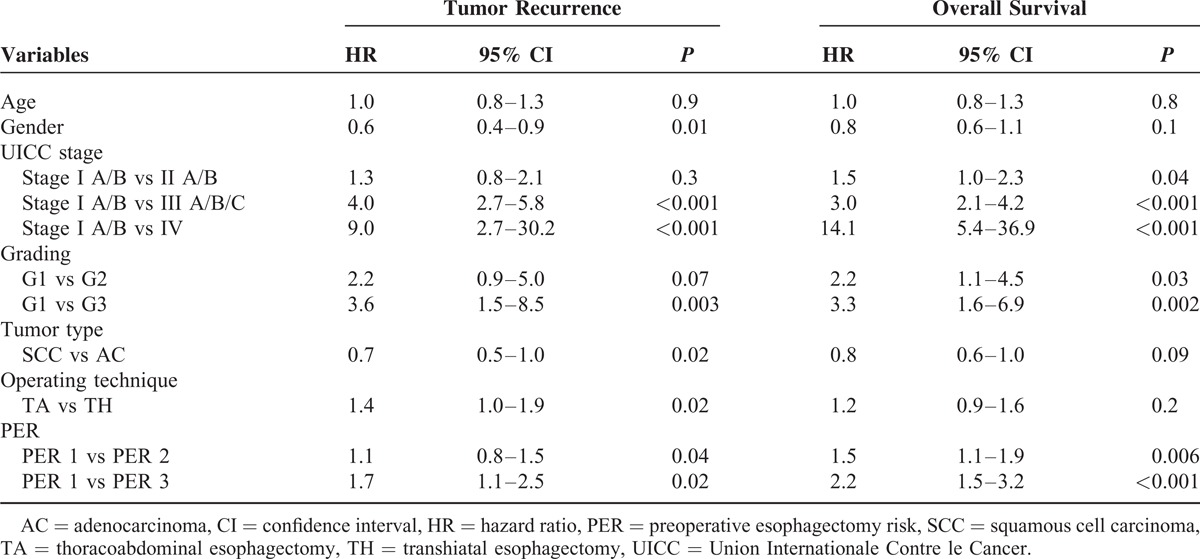
Multivariate Cox Regression Analysis for Tumor Recurrence and Overall Survival

The multivariate analysis was repeated after stratification of the underlying histology and operating technique was performed. However, the PER score, besides tumor size and lymph-node and distant metastases, remained throughout an independent prognostic factor of survival in EC patients. In the subanalyses, AC patients had an HR of 1.7 (CI: 1.3–2.1, *P* < 0.001) and SCC patients had an HR of 1.4 (CI: 1.1–1.6, *P* = 0.005) for death with increasing PER score. A similar tendency was seen in the subanalyses for operating technique. Patients undergoing TA resection had an HR of 1.5 (CI: 1.2–1.8, *P* = 0.001) and patients with TH resection had an HR of 1.5 (95% CI: 1.2–1.9, *P* < 0.001) for death with increasing PER score.

## DISCUSSION

A clinically useful risk score should be easy to perform and be based on a simple diagnostic work-up. Basic diagnostic work-up for all types of major surgery includes assessment of the cardiopulmonary system and blood testing for evaluation of inflammation and hepatorenal function. Only a few of the published risk scores were able to predict mortality, and most of them failed to predict the morbidity or oncological outcome of EC patients.^5,7,15–17^ Furthermore, reported scores in the literature were not easy to determine due to the requirement of special tests or the need for complex algorithms. Considering the high mortality and morbidity rates associated with esophageal resection, there is an urgent need for preoperative risk stratification. Previously, Bartels et al described a risk score for the prediction of mortality with esophageal surgery.^18^ The Bartels score, however, requires an aminopyrine breath test to assess the hepatic function. Besides the complexity of this test, such a tool is not widely available. Furthermore, the Bartels score includes the Karnofsky index, and evaluation of the cardiac status is done without validated tools. The Karnofsky index is a widely used tool to evaluate the general condition of the patient but lacks objectivity. Importantly, the Bartels score is limited to predict only mortality. The POSSUM tool was developed to predict mortality and morbidity in patients undergoing major surgery and has been modified to O-POSSUM for utility in esophageal surgery.^6,19^ Apart from the extensive testing required to determine the O-POSSUM score, it failed to predict the perioperative mortality and morbidity in esophageal surgery.^7^

The PER score enabled risk stratification of perioperative morbidity and mortality in patients undergoing esophageal resection. The prognostic value of the PER score was not limited to mortality and morbidity but also represented an independent prognostic factor of overall oncological outcome.

The PER score reported in this study is based on validated tests and has been verified in a large, homogeneous study population. The uniqueness of the PER score is its simplicity because blood testing, survey of medical history, and pulmonary function testing represent the minimum diagnostic work-up necessary for esophageal surgery. In our model, cardiac risk is assessed by the RCRI, which has been validated for the prediction of operative risk in cardiac and noncardiac surgery and thus has become 1 of the most widely used risk indices.^8,20–23^ The PFT represents an objective test, and risk stratification is based on stringent values. The MELD score is currently considered the gold standard for the urgency of liver transplantation but has also been validated in nonhepatic surgery for mortality and morbidity.^9–11^ Based on these tools, the PER score can easily be calculated independent of individual assessment.

A potential bias of neoadjuvant treatment does not apply to our study population since none of our patients received preoperative chemotherapy or radiochemotherapy. Previously reported data have shown a close relationship between neoadjuvant chemoradiotherapy with increased mortality and esophageal surgery.^24^

Lagarde et al^7^ postulated the urgent need for future predictive models of EC surgery to focus not only on perioperative outcome but also on oncological survival. Until now, no score has been reported being able to predict mortality, morbidity, and oncological outcome for EC. The most commonly used predictive parameters for long-term survival are tumor-specific parameters like tumor size, presence of lymph-node or distant metastases, or grade of differentiation. However, there is evidence that factors not apparently associated with the tumor, like inflammation, do correlate with the oncological outcome. The Glasgow prognostic score (GPS) has been shown to be an independent prognostic factor for survival in colorectal cancer and other kinds of cancer.^25–27^ Kobayashi et al^28,29^ reported on the prediction value of GPS in neoadjuvantly chemoradiotherapy-treated SCC patients. GPS is based on the determination of C-reactive protein and albumin as markers for inflammation. The significance of GPS has been verified by several groups^30^ and has been reported to be superior to blood-tumor markers like carcino-embryonic antigen (CEA) and carbohydrate antigen 19-9 (CA 19-9) in colorectal cancer.^31^ In concordance to this the herein reported PER score also classified patients into 3 different groups based on preoperative risk assessment. Tumor biology may affect cardiac, pulmonary, and liver function. Another possible explanation could be that patients with less cardiac, pulmonary, or liver function are not able to react adequately or sufficiently on tumor growth and metastatic spread. Both potential explanations cannot be proven by our findings and have to be addressed in future studies.

A close relation between the presence of lymph-node metastases and the PER score and the inclusion of M1 patients in our study may be considered a bias because of the close association of OS and the score. However, we were able to show the PER score remained a significant prognosticator of OS in EC patients after stratification of the presence of lymph-node and distant metastases.

Although this is the first attempt to predict postoperative outcome of esophageal surgery based on objective preoperative parameters, a short coming of our study is the lacking validation by other centers for esophageal surgery, which mandates further verification and validation of this risk score in future studies.

In conclusion, the PER score is an easy-to-determine and an objective tool. The score seems to be superior to other reported prediction models and normograms for esophageal surgery. Therefore, adoption of the PER score as a simple and convenient tool not only for the prediction of perioperative outcome in terms of mortality and morbidity but also of long-term survival in patients undergoing esophageal resection for EC, independent of underlying histology and operating technique, may be helpful to stratify patients hierarchically into different risk groups preoperatively. Thus, multicenter studies are needed for independent validation of the PER score.
